# Dangguijagyag-san for primary dysmenorrhea: A protocol for systematic review and meta-analysis of randomized controlled trials

**DOI:** 10.1097/MD.0000000000018345

**Published:** 2019-12-16

**Authors:** Jihye Seo, Donghun Lee, Hee-Geun Jo

**Affiliations:** a Chung-Yeon Korean Medicine Hospital, Seo-gu, Gwangju; b College of Korean Medicine, Wonkwang University, Iksan, Jeonbuk; c Department of Herbal Pharmacology, College of Korean Medicine, Gachon University, Sujeong-gu, Seongnam; d Chung-Yeon Central Institute, Seo-gu, Gwangju, Republic of Korea.

**Keywords:** Danggui Shaoyao San, Dangguijagyag-san, herbal medicine, primary dysmenorrhea, systematic review

## Abstract

**Background:**

Primary dysmenorrhea is the most common gynecological disorder in women of reproductive age. In East Asia, traditional herbal medicines have been used for a long time to treat symptoms of primary dysmenorrhea. Dangguijagyag-san (DJS) is one of the most widely known traditional herbal medicine for primary dysmenorrhea. Although there was the previous systematic review of DJS, it had some limitations. To assess the effectiveness of DJS for primary dysmenorrhea and to update the previous review, this protocol was developed to conduct a systematic review and meta-analysis.

**Methods:**

We will search the randomized controlled clinical trials of DJS for primary dysmenorrhea from inception to April 2019. The search database will be PubMed, EMBASE, Cochrane Central Register of Controlled Trials, Oriental Medicine Advanced Searching Integrated System, Korean Traditional Knowledge Portal, Korean Medical Database, National Digital Science Library, and the China National Knowledge Infrastructure. Our 2 authors will perform the selection of studies, the extraction of data, and the quality assessment with risk of bias tool independently. To analyze the data, we will conduct the quantitative synthesis.

**Results:**

We will synthesize the data from selected studies and estimate the strength of the evidence DJS for the treatment of primary dysmenorrhea.

**Conclusion:**

This study will provide the scientific evidence of DJS.

**Systematic review registration:**

PROSPERO registration number is CRD42019130768.

## 1 Introduction

Primary dysmenorrhea is a gynecologic disorder characterized by spasmodic cramping and pain in the lower abdomen, just before and/or during menstruation.^[1]^ The pain usually lasts for 8 to 72 hours, often accompanied by systemic symptom such as insomnia, headache, fatigue, nausea, or vomiting.^[1]^ Prevalence of dysmenorrhea has been reported in the range between 16% and 91% of women of reproductive age and 2% to 29% of severe pain.^[2]^ In the case of primary dysmenorrhea, unlike secondary dysmenorrhea, identifiable pathological findings such as endometriosis, adenomyosis, and so on are not identified.^[3]^ For this reason, although primary dysmenorrhea is the most common gynecological disorder, it is often regarded as a normal part of the menstrual cycle and remains a poorly understood disorder from a scientific point of view.^[4]^

Pathophysiology of primary dysmenorrhea is known to be closely associated with overproduction of uterine prostaglandin.^[5]^ Increased release of prostaglandin leads to myometrial hypercontractility, resulting in ischemia of the uterine muscle, leading to dysmenorrhea pain. In general women, prostaglandin levels increase during the luteal phase compared to the follicular phase. However, studies show that prostaglandin levels measured by endometrial biopsy during the luteal phase in women suffering from dysmenorrhea are higher than enumenorrheic women.^[5]^ Exogenous prostaglandin also induces nausea and diarrhea, which are common synchronous symptoms of dysmenorrhea. Therefore, several clinical trials aimed at the relief of multiple dysmenorrheic symptoms focus on the inhibition of prostaglandin. At present, the most frequently prescribed drugs used in the treatment of dysmenorrhea based on the prostaglandin pathology of primary dysmenorrhea is nonsteroidal anti-inflammatory drugs (NSAIDs), prostaglandin synthase inhibitors.^[6,7]^ If women suffering from dysmenorrhea do not respond to prostaglandin inhibitors, synthetic hormones in oral contraceptive may be administered to reduce prostaglandin synthesis and suppress dysmenorrheic pain.^[3]^ However, such conventional medicines have disadvantages in terms of safety. In the case of NSAIDS, various side effects on liver, kidney, and digestive tract have been reported, and oral contraceptive has also been identified as a risk of venous thromboembolism in long-term use based on recent meta-analysis results.^[8,9]^ In addition, there is still a need for further research on nondrug treatments that can be widely used instead.^[10]^ Given this situation, Traditional East Asian herbal medicine can be a significant alternative.

In East Asia, herbal medicine has long been used as a safe and effective intervention for the treatment of primary dysmenorrhea. Recent studies have confirmed that herbal medicine formulas commonly used in primary dysmenorrhea exert analgesic effects by mechanisms such as anti-inflammation, reducing prostaglandin, and vasorelaxation.^[11,12]^ Among these herbal medicines, Dangguijagyag-san (DJS) is one of the most widely known traditional herbal formulas for primary dysmenorrhea. In recent studies, DJS has been shown to have effects such as suppression of uterus contraction, prostaglandin level reduction, and correction of luteal insufficiency, demonstrating the potential of promising drugs for primary dysmenorrhea.^[13–15]^ DJS has also demonstrated analgesic effects on primary dysmenorrhea in double-blind clinical trials.^[16]^

To evaluate the clinical efficacy of DJS that is experimentally evident for primary dysmenorrhea, a comprehensive systematic review and meta-analysis is required. This study will be helpful clinicians to select DJS for primary dysmenorrhea as alternative treatment. Although the previous systematic review of DJS has shown the effects for primary dysmenorrhea, there were some limitations in terms of the fact that the statistical heterogeneity was high and subgroup analysis was not performed.^[17]^ Our study will include the subgroup analysis to investigate heterogeneous results.

The most recent systematic review study included randomized controlled clinical trials (RCTs) up to 2015.^[18]^ As we identified several RCTs have been published since 2016, it is thought that systematic review including these studies is necessary.^[19,20]^ This protocol was developed to update a systematic review and meta-analysis of DJS for primary dysmenorrhea.

## 2 Methods

### 2.1 Study registration

We registered the protocol on PROSPERO (registration number: CRD42019130768). This protocol is developed according to the preferred reporting items for systematic reviews and meta-analysis protocol (PRISMA-P).^[21]^ When we perform this systematic review, we will follow the Cochrane Handbook for Systematic Reviews of Interventions and the PRISMA guidelines.^[22,23]^ If there are differences with this protocol in the full systematic review, we will update the changes in our full review.

### 2.2 Data search methods

We will electronically search the articles in the PubMed, EMBASE, and Cochrane Central Register of Controlled Trials (CENTRAL). We will also search in the following Korean and Chinese databases: 4 Korean medical databases (Oriental Medicine Advanced Searching Integrated System, Korean Traditional Knowledge Portal, Korean Medical Database, and National Digital Science Library (NDSL)), and 1 Chinese database (the China National Knowledge Infrastructure). The article search in each database will be performed from their inception to April 2019.

We will include the terms about primary dysmenorrhea and DJS in the searching terms. To perform a comprehensive search, we will develop the search strategy using the related term. The example search strategy in Table 1 will be used for CENTRAL.

**Table 1:**
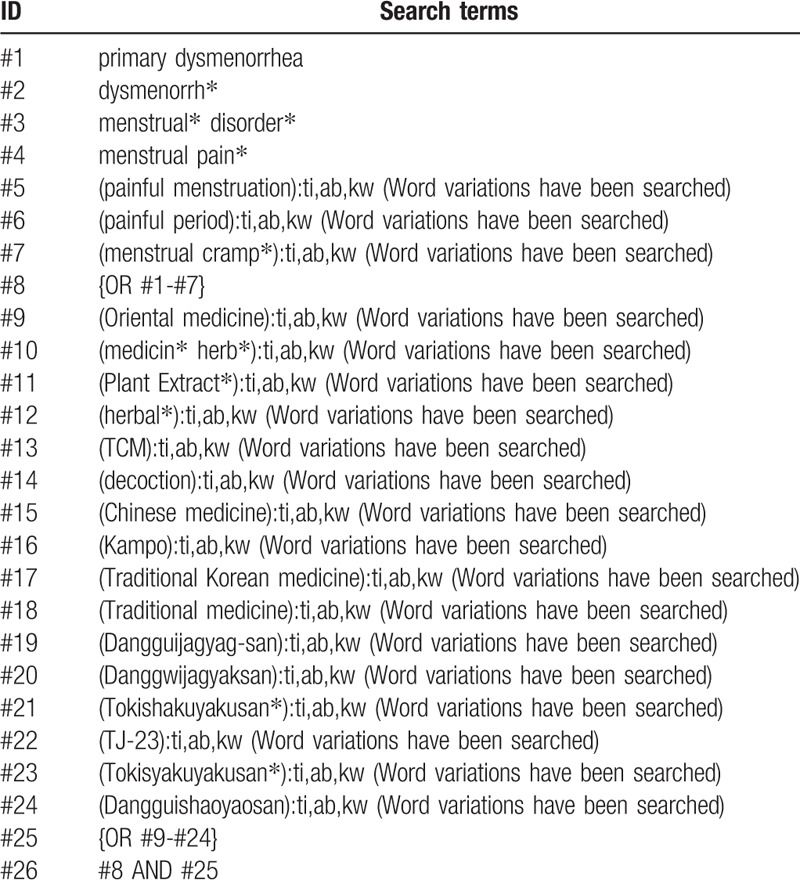
The search strategy for Cochrane Central Register of Controlled Trials.

### 2.3 Inclusion criteria

#### 2.3.1 Study type

We will include the studies which are RCTs including quasi-RCTs. Controlled (nonrandomized) clinical trials will be excluded. Considering the research environment, the included study will be limited to the literature written in English, Korean, or Chinese.

#### 2.3.2 Study object

We will include the primary dysmenorrhea patients who have menstrual pain in the absence of pelvic pathology. Regardless of the sex and age, we will include all participants.

#### 2.3.3 Intervention type

In the experimental group, the interventions of included study are DJS alone or as a combination with other treatments. We will include DJS and modified DJS, regardless of the formulation. In the control group, we will include the study using western medicine, placebo, or the other herbal medicine as a control intervention. In the case of the other combination intervention, we will include the study that the control group received the same treatment as the intervention group as adjunctive treatments.

#### 2.3.4 Primary and secondary outcomes

We will investigate the change of menstrual pain intensity and the total treatment response rate as the primary outcomes. The pain intensity is indicated by the visual analog scale or the numerical rating scale. Secondary outcomes will include the use additional analgesics, overall related symptoms, and the quality of life measured with validated questionnaires. We will also include the adverse events as the secondary outcome.

### 2.4 Study selection

**Figure 1:**
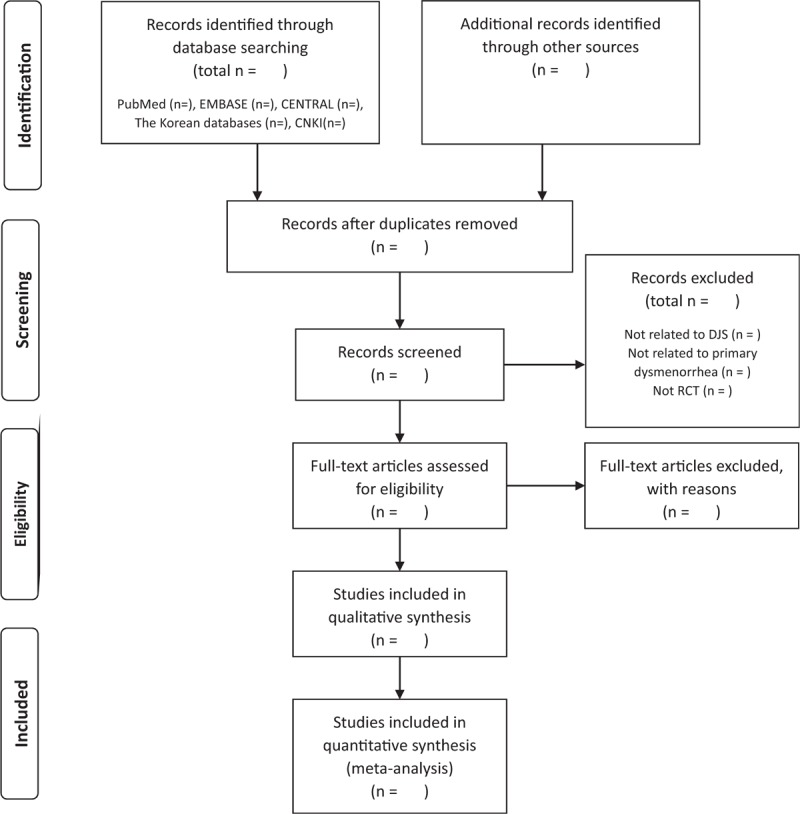
The flow chart of the study selection process. CENTRAL = Cochrane Central Register of Controlled Trials, CNKI = Chinese National Knowledge Infrastructure, DJS = Dangguijagyag-san, RCT = randomized controlled trial.

In study selection process, we will use the Endnote referencing software. The results of searching process will be exported to the Endnote. Duplicate studies will be removed using this software. Two authors will perform study selection independently. Two authors will discuss the way to select studies before performing selection process, and if there are any disagreements in this process, we will discuss and resolve the different selection. In the first stage of selection, we will evaluation the titles and abstracts of studies and select those likely to be of relevance to our review. After first selection, we will go over the full-text of the selected studies and confirm the appropriate studies for our review. We will write and summarize this study screening and selection process in the PRISMA-compliant flow chart. The result of study selection and the reasons for excluding studies will be presented in the PRISMA flow chart (Fig. 1).

### 2.5 Data extraction and quality assessment

Two independent authors will carry out the data extraction and the quality assessment. The authors will progress preliminary this process. To perform the extraction and quality assessment, we will use the Excel program and the extraction and assessment form will be designed in consensus of all authors. We will extract the data including the year of study publication, participant characteristics, the number of participants, dropouts, study period, intervention details, outcomes, and adverse events. To assess the quality of the studies, we will use the “risk of bias” tool from the Cochrane Handbook V.5.1.0. We will determine the bias including the following items: random sequence generation, allocation concealment, blinding of the participants and personnel, blinding of the outcome assessments, incomplete outcome data, selective reporting, and other sources of bias.

When the studies contain insufficient data to extract or assess, we will contact the authors by e-mail to request the necessary data. Two authors will cross-check the results and any disagreement between the results will be resolved with a discussion. If there is disagreement that cannot be resolved with a discussion, it will be resolved by the other authors.

### 2.6 Data analysis

To analyze the extracted data, we will use the Cochrane Collaboration's software program Review Manager (RevMan version 5.3) for Windows. When selected studies are sufficient, we will conduct the quantitative synthesis. When we conduct the quantitative synthesis, we will synthesize each outcome from the homogeneous studies. We will include each participant's outcome measurement only once and perform the data analysis with the values that were measured at the end of the treatment period. We will use fixed or random-effects model considering the type of outcomes to perform the quantitative synthesis with the RevMan program. For synthesize dichotomous data, we will calculate the date and present it by the relative risks with 95% confidence intervals (CIs). If the outcome is continuous data, we will calculate the effect size using the mean differences with 95% CIs. For the homogeneous outcome presented by different scales, we will calculate the effect size using the standard mean difference.

When homogeneous studies are not sufficient to quantitative synthesis, we will analyze the data qualitatively. If we could estimate the strength of the evidence, the results of the data analysis will be assessed with the grading of recommendations assessment, development, and evaluation.

The heterogeneity between the outcomes from studies will be assessed by the *I*^2^ statistic value calculated with the RevMan program. If the calculated *I*^2^ is more than 70, we will judge that the heterogeneity is high. When heterogeneity is high, we will investigate the possible causes of the heterogeneity and perform subgroup analyses or explain the cause.

We will assess the reporting biases with a funnel plot, when included studies are sufficient (at least 10 studies). When there are the reporting biases, we will explain the possible reasons for asymmetries in reporting.

#### 2.6.1 Subgroup analysis

We will conduct subgroup analysis according to the different combinations of interventions. If the heterogeneity is high, we will also consider to perform a subgroup analysis. To investigate the possible causes of high heterogeneity, we will review the quality of the study, patient characteristics (severity and symptoms) or the other factors affecting the outcomes.

For subgroup analysis, detailed considerations will be as follows:

(1) Patient characteristics: severity, duration or the differentiation of syndromes
(2) Types of DJS: decoction, tablet or powder.
(3) Control interventions: western medicine, placebo, no treatment or the other herbal medicine.
(4) Combination intervention: depending on the type of combination therapy involved.

## 3 Discussion

Primary dysmenorrhea patients are treated by NSAIDs as first-line treatment, but NSAIDs have gastrointestinal side effects and ceiling effects.^[24,25]^ As complementary or alternative therapies, acupuncture and local heat patches are also effective.^[3]^ In clinical practice in East Asia, including China and Korea, herbal medicines composed according to traditional prescription have been commonly used to treat primary dysmenorrhea.

Several meta-analyses have been conducted to assess the efficacy of herbal medicines or Chinese herbal medicines that were composed of many other herbs.^[17,26,27]^ DJS is one of the effective herbal medicine prescriptions used for dysmenorrhea.^[28]^ Although the efficacy and safety of DJS was evaluated in previous reviews, the quality of evidence was low and the heterogeneity was high.^[18]^ We examined the reasons for the high heterogeneity and found that one of the included studies was a combination intervention. To explore the high heterogeneity in previous studies, we will conduct subgroup analysis according to the different studies’ characteristics. For planning subgroup analysis, we also reviewed the relating studies and found RCT including the primary dysmenorrhea patients with insufficiency of qi and blood symptoms according to the theories of traditional medicine. In this study, DJS was more effective in the patients with insufficiency of qi and blood symptoms than the other herbal medicine formulas.^[29]^ Therefore, unlike previous studies, we will include other herbal medicines in the control group which may provide additional evidence for DJS prescribing differently according to the symptoms and personal characteristics in clinical practice. In addition, we will search recent RCTs and update the evidence of DJS.

The results of our study will provide updated evidence of DJS and helpful information for clinicians and patients to treat primary dysmenorrhea.

## Author contributions

JS: developed search strategy and wrote the protocol

DL: revised the manuscript

HGJ: revised and examined the protocol

All authors have read and approved the final manuscript.
